# Hypertension as a factor associated with hearing loss

**DOI:** 10.1016/S1808-8694(15)31001-6

**Published:** 2015-10-19

**Authors:** Luciana Lozza de Moraes Marchiori, Eduardo de Almeida Rego Filho, Tiemi Matsuo

**Affiliations:** a PhD in medicine and Health Sciences, Professor of Speech Therapy and Pharmacy - Norte do Paraná University; b PhD in Medicine, Professor of the Postgraduate course in Medicine and Health Sciences – Londrina State University; c PhD in Statistics, Professor of the Postgraduate course in Medicine and Health Sciences – Londrina State University. Universidade Norte do Paraná (UNOPAR)

**Keywords:** risk factor, hypertension, hearing loss, quality of life

## Abstract

**Aim:**

To identify likely association between blood hypertension and hearing loss. Design: A non-paired case-control study. Setting: Institutional work carried out at Universidade Norte do Paraná, in South Brazil.

**Material and Method:**

154 cases and 154 controls, both genders, aged 45 to 64, included in the research after sample estimation. Methodology: Hypertension was verified through blood pressure readings and by a systematized questionnaire about hypertension and the use of medication for blood pressure. Hearing was assessed through tonal threshold audiometrics and audiologic anamneses. Non-conditional logistic regression was used in order to control likely confusion or modification of effect of other variables on interest associations.

**Results:**

There is a significant association between blood hypertension and hearing loss. Hearing loss in the population under study suggests that hypertension is an accelerating factor of degeneration of the hearing apparatus due to aging.

**Conclusions:**

The results in this research, through evidence of association between blood hypertension and hearing loss, can allow for an integrated work of cardiologists, nephrologists, otorhinolaryngologists, audiologists and other health professionals concerned with alterations caused by blood hypertension.

## INTRODUCTION

Most adult acquired hearing loss have gradual onset and may impair oral language reception.

According to data from the ASHA (American Speech - Language - Hearing Association)^1^, there are currently 28 million individuals in the USA with some type of hearing loss, and 80% of those are irreversible cases. These data also show that 4.6% of the individuals between 18 and 44 years have hearing loss, while 14% of middle aged individuals – between 45 and 64 years, and 54% of the population above 65 years have some hearing loss. This is due to a number of factors, such as intense and/or continuous noise exposure, inhalation of toxic substances, ingestion of ototoxic drugs, metabolic and circulatory alterations, infections, different types of injuries and genetic inheritance.

The relevance of Arterial Hypertension as a human disease is due to its clinical complications, morbidity and mortality, as well as the heavy burden to the patient, family and public coffers. Few diseases are responsible for so frequent and severe complications as the ones accruing from Arterial Hypertension: stroke, heart, kidney and peripheral vascular insufficiencies. Moreover, it is estimated that about half of the deaths of patients above 50 years are due to cardiovascular diseases, and 80% of them have high blood pressure^2^.

The size of this “High Blood Pressure” situation in Brazil is estimated through rates established on international papers, and based on projections from the Brazilian Institute of Geography and Statistics Census of 1990, there are today about 90 million Brazilians aged above 20 years. If we consider the hypertension prevalence to be 20%, there must be at least 18 million hypertensive Brazilians, of which about 50% are not aware they have the disease^2^.

Katz^3^ says that all living cells in the human body depend on a proper supply of oxygen and nutrients in order to maintain their function, and such supply depend on the functional and structural integrity of the heart and blood vessels. Hypertension, the most common vascular disorder, may facilitate structural changes in the heart and blood vessels^4^. High pressure in the vascular system may cause inner ear hemorrhage, which is supplied by the anterior inferior cerebelar artery, which supports the inner ear artery and is divided into cochlear artery and anterior vestibular artery^5^, which may cause progressive or sudden hearing loss^3^, ^4^. This circulatory system pathology may directly affect hearing in a number of ways. One of the vascular physiopathological mechanisms described is the increase in blood viscosity, which reduce capillary blood flow and ends up reducing oxygen transport, causing tissue hypoxia, thus causing hearing complaints and hearing loss in patients^6^. Moreover, arterial hypertension may cause ionic changes in cell potentials, thus causing hearing loss^7^.

Since the second half of last century, studies have analyzed if individuals with arterial hypertension have more hearing loss when compared to other individuals. However, results have been unclear, and there is the need of further studies in this area to prove if there really is a prevalence increase of such alteration as far as peripheral and central hearing are concerned in the affected population.

Initially, Rosen et al.^8^ stated that in a study carried out with hypertensive patients in the USA, there was a correlation between high blood pressure and hearing loss in high frequencies. However, such correlation was not seen by the same authors in a later study carried out with a Sudanese native population. Hansen et al.^9^, in a retrospective study carried out in Denmark with the charts of 342 patients assessed between 1945 and 1961, consecutively, do not relate arterial hypertension to hearing loss in this population.

Marková^10^, in the Tcheck Republic, after analyzing the hearing symptoms of 50 hypertensive patients submitted that arterial hypertension is an important risk factor for hearing loss.

Nazar et al.^11^, in a study carried out with controlled chronic hypertensive patients in specialized centers, without diabetes and without exposure to intense noise and ototoxic drugs for at least 3 years, observed that of the 217 controleld chronic hypertensive patients, most presented with hearing alterations; however, with varied audiogram profiles. After such study, the authors stated that individual predisposing factors (structural or metabolic) may, in isolate cases, cause hearing loss, which does not represent a habitual development in chronic hypertensive patients.

Brohem et al.^12^ assessed audiometrically 50 hypertensive patients with ages above 45 years in Brazil, and 62% of those had sensorineural hearing loss.

In a controlled-case study carried out in Kenya with 50 elderly individuals using ABR, Chen et al.^13^ observed a relationship between hearing loss and arterial hypertension in this population.

Marchiori et al.^14^, aiming at observing the frequency range and the audiometric profile of hypertensive individuals, studied 552 audiologic exams from patients referred to audiologic assessment. Of the 552 exams studied, 137 were from patients with arterial hypertension of both genders, with ages varying between 14 and 84 years, and 121 (88.32%) of these hypertensive patients had hearing loss, most of them (43.06%) were moderate, sensorineural (38.32%), and/or had hearing complaints such as tinnitus, fullness in the ear and vertigo.

Rey et al.^15^, tested 59 patients with mean age of 75 years and noticed a significantly negative relationship with hypertension.

Amstutz-Montadert et al.^16^, reported that in cases of early presbyacusia, around 55 years of age and of fast evolution, metabolic or vascular disorders, use of ototoxic drugs, nicotine and exposure to noise must be investigated, since they all contribute to worsen cases of presbyacusia.

Since there are not too many papers in the literature that may lead us to strong evidence, our paper aims at checking if there are associations between arterial hypertension and hearing loss in individuals with ages ranging between 45 and 64 years.

The results attained with the present research project will certainly serve as basis for a greater integration between cardiologists, nephrologists, otorhinolaryngologists, speech therapists and other health care professionals involved with arterial hypertension and hearing loss care, bringing about relevant data for the professionals involved in order to improve quality of care in the therapy and rehabilitation of these patients.

## METHODS

This research was carried out through a case-controlled study, after the project was analyzed and approved, and an informed consent was signed, pertaining to resolution 196/96-CNS, for the duration of the project, that extended to 2004.

308 middle aged individuals were part of the research (aged between 45 and 64 years). The 154 patients with hearing loss (incident cases) were obtained in the sequence of medical visits, while the 154 individuals without hearing loss (controls) were recruited by the patients themselves, under the instruction that they had to live close to the patients they were matching and could not be siblings or parents, in order to avoid genetic biases regarding both Arterial Hypertension and Hearing loss.

We excluded patients with previous history of: specific hearing disorders (such as rubella and head injuries), specific metabolic disorders (such as diabetes) and specific vascular disorders (such as strokes), and also individuals who worked or had worked in an environment that could cause noise-induced hearing loss, patients with kidney diseases and with prior history of hospital stay or ingestion of potentially ototoxic medication or drugs were also taken off the study.

The major variable of exposure in our study was Arterial Hypertension, and the outcome variable was hearing loss.

The instrument for hearing assessment was an audiologic interview which is routinely used in the department of Audiology - UNOPAR, based on the interview protocol (Katz^3^) and the tonal threshold audiometry considered a gold standard for the evaluation of the auditory threshold in adults^17^, written in the tonal audiometry form used in the routine audiologic visit at UNOPAR and passed in the Winaudio database in order to be stored and printed for the patient.

In order to assess blood pressure we used a tool for systematized clinical data filling out and some questions:

Identification data: name, age, gender, schooling.

Weight, height, arterial pressure.

Questions:

1. Do you have high blood pressure?
  ( ) yes ( ) no
2. Have you had your blood pressure measured recently?
  ( ) yes ( ) no
3. When was the last time you measured your blood pressure?
4. What has been your blood pressure recently? SP X DP
5. Which physician or Health Care facility controls your blood pressure?
6. Do you usually take medication to control your blood pressure?
  ( ) yes ( ) no
7. Which medication (s) do you take?

Blood pressure was measured indirectly with a proper cuff and mercury gauge - Tycos®. We respected the guidelines established by the III Brazilian Consensus on Hypertension of 1998, which are equivalent to the standards proposed by the European Society of Hypertension^18^, ^19^.

Patients with arterial blood pressure ≥ 140 × 90mm of Hg in different measures, and those who answered yes to questions 1 and 6 and identified the medication(s) they were using were considered positive for high blood pressure.

We noticed this association between hearing loss and each one of the variables in a first stage (raw analysis), through the attainment of an odds ratio estimate per point and per range, besides the Mantel-Haenszel chi-squared statistical value; and the significance of variables such as smoking, alcohol intake, physical exercises, gender, age, BMI (body mass index) and schooling were all considered. In the next stage we selected the variables that had significant values for the descriptive level. Non-conditional logistics analysis (since there was no pairing), was used in order to attain the odds ratio for hearing loss and to help investigate the existence of interactions between the variables, controlling the effects of the previously selected factors. The statistical modeling process was carried out using the Stepwise selection method.

The sample size was calculated using the Epi 6 software, considering data from a pilot study developed with the population in question, in which the ratio of hearing loss and high blood pressure individuals was defined (37.7%) as was the ratio of individuals without hearing loss and high blood pressure (18.8%), with age varying between 45 and 64 years. With an odds ratio of 2.63 and a power of 95% the total N of participants was estimated to be 308.

## RESULTS

As to the complete distribution of the cases and controls according to hypertension and hearing loss, we saw that 72 individuals had hearing loss and high blood pressure, 82 had hearing loss and no normal blood pressure, 46 did not have hearing loss but had high blood pressure and 108 did not show hearing loss, nor high blood pressure. (Table 1).

**Table 1:** Complete distribution of cases and controls according to high blood pressure and hearing loss.

Arterial Hypertension	HEARING LOSS			
	Yes		No	
	n	%	n	%
YES	72	46,8	46	29,9
NO	82	53,2	108	70,1
TOTAL	154	100,0	154	100,0

OR = 2,06[1,26 < OR < 3,39]; X^2^ = 8,59 (p = 0,0034)

The cases presented average age in years of 56.1, a little higher than the 52.8 presented by the control group.

There were more women among the cases 57.8%, and among the controls 77.3% when compared to men.

We could notice that, regarding schooling, all the groups presented higher incidence of individuals with first grade education completed, and among individuals with hearing loss and hypertension the incidence was 30.6%; among individuals with hearing loss and no high blood pressure, the incidence was 23.5%, among individuals without hearing loss and high blood pressure the frequence was 50% and among individuals without hearing loss and without high blood pressure, the frequence was 26.9%.

Most individuals in this research did not smoke; among the individuals with hearing loss and high blood pressure, 84.7% did not smoke. Among individuals with hearing loss and without high blood pressure, 91.3% did not smoke and among the group without hypertension and without hearing loss, 83.3% did not smoke (p= 0.5920).

Most individuals in this research did not drink alcoholic beverages; the group of individuals with hearing loss and hypertension, 94.4% said they did not ingest alcohol; in the group with hearing loss and without arterial hypertension, 96.3%; in the group without hearing loss and with arterial hypertension, 100%; and in the group without arterial hypertension and without hearing loss, 94.4% reported the same (p= 0.4206).

As to the practice of physical exercises, in the group of individuals with hearing loss and arterial hypertension, 63.9% reported they did not practice any type of exercise; in the group of patients with hearing loss and without arterial hypertension, 65.9%; in the group without hearing loss and with arterial hypertension, 67.4%; and in the group without arterial hypertension and without hearing loss, 68.5% reported the same (p=0.9298).

As to the type of hearing loss in the sample, the individuals with arterial hypertension and those without arterial hypertension presented a similar frequence of sensorineural hearing loss, and in the first group, 83.7% of the ears presented the aforementioned loss, while in the second group, 81.8% had it (p=0.1310).

As to the degree of hearing loss in the sample, the individuals with arterial hypertension and those without it had a similar frequence of mild hearing loss, in the first group this rate was of 62.0% and in the second group 63.6% (p=0.0881).

The arithmetic average of body mass index in the group of individuals without hearing loss and with arterial hypertension was of 30.3 and in the group with hearing loss and arterial hypertension it was of 29.7; while the group with hearing loss and without arterial hypertension this value was of 27.6 and in the group without hearing loss and no hypertension this number was of 26.8, showing that in this study there was a higher body mass index in the individuals with arterial hypertension.

As to the use of anti-hypertensive medication, we noticed that of the 72 cases with hypertension, only 5 (6.9%) did not use the medication, and of the 46 controls who had hypertension, only one (2.1%) did not use medications; thus, of the 118 hypertensive individuals in this study, only 6 (5%) did not use medication to treat their hypertension at the time of the exam.

As to the association of antihypertensive drugs, 24 cases (33.3%) and 11 controls (23.9%) used more than one anti-hypertension drug, which prevailed among cases - 6 individuals (8.3%) and among the controls - 5 individuals (10.8%), with association of diuretic agents plus ACE inhibitors.

Among the cases, 34 (47.2%), and among controls, 17 (36.9%) used ACE inhibitors.

Among the cases, 25 individuals, and among controls, 14 used diuretic agents, 17 of the cases and 15 of the controls used β-blockers and one of the cases and 3 controls used angiotensin II antagonist inhibitors; 8 of the cases and 2 controls used calcium channel blockers; 3 of the cases and 1 control used α-blockers; and 4 cases and 5 controls used other anti-hypertension medications such as cloridine, α-metildopa and minoxidil and, 7 cases and 4 controls were using other drugs simultaneously such as antiarrhythmics, vessel dilators and antiplatelet aggregation drugs.

Arterial hypertension, advanced age and male gender proved to be independent risk factors for hearing loss (Table 2).

**Table 2:** Results of the logistics analysis for hearing loss with independent variables.

Risk factors	Odds ratio	I.C.I.	95%	Coefficient	Standard error	Wald test	P value
AH (Y/N)	1,7277	1,0475	2,8496	0,5468	0,2553	2,1419	0,0322
AGE (high/low)	2,2499	1,3822	3,6620	0,8109	0,2486	3,2622	0,0011
GENDER (Male/Fem)	2,5444	1,5244	4,2470	0,9339	0,2614	3,5730	0,0004
Total	*	*	*	-0,1364	0,2189	-0,6230	0,5333

The logistic regression model shows that hypertension (p=0.0322), higher age (p=0.0011) and male gender (p=0.0004) are independent risk factors for hearing loss.

## DISCUSSION

The present study showed the existence of an association between hearing loss and arterial hypertension in individuals between 45 and 64 years, and contributed with such evidence to explain this controversial aspect of human health. Such association between hearing loss and arterial hypertension has been an important object of research in recent decades, with highly antagonic conclusions, and some authors back this association^8^, ^9^, while others deny it and even present different results in studies carried out at distinct times^8^, ^9^, ^10^, ^11^, ^16^, ^17^, ^18^, ^19^, ^20^.

As to the methodological characteristics of this study, the care taken in outlining the age factor, focusing on the age range of middle aged individuals, between 45 and 64 years as they do in hypertension investigations^22^, ^23^, the strict exclusion criteria, eliminating individuals with diseases and specific activities capable of producing hearing alterations and the care taken in diagnosing their hearing loss and arterial hypertension certainly helped to reduce selection biases. Notwithstanding, memory biases during history taking, use of ototoxic medication may have affected the results; notwithstanding, in studies about information reliability, for example regarding metabolic and vascular alterations^24^, or of information biases, for example regarding metabolic and vascular alterations biases yet undiagnosed or not reported by the patient.

Pertaining to the statistical analysis, we used logistics regression since this is a model that with a good adjustment obeys the principle of sobriety, and it also describes the relation between one result and a set of simultaneous explanatory variables, since the logistics analysis controls a great number of variables at the same time, allowing the data to be used in a more efficient way^25^. In this study we could see a certain uniformity among the results obtained with the application of the logistics regression analysis that became practically mandatory since it has a greater power for exploring the variables in question.

With aging, there is a higher number of chronic diseases. Systemic arterial hypertension and hearing loss have important prevalence in the elderly population^20^. In this paper we observed that although the sample individuals were between 45 and 64 years (middle aged), the higher age range (p=0.0011) proved to be an independent risk factor for hearing loss when we consider the logistic regression model used. This is probably due to the fact that, as we all know, with age there are structural alterations in the whole body, including the hearing system^1^.

Despite these structural changes caused by age, many authors mention presbyacusia, which usually start at around 65 years of age and is a hearing loss type accruing from aging itself and is associated to specific audiologic characteristics, being a descending, bilateral and symmetrical sensorineural hearing loss type^1^, ^2^, ^3^, ^4^, ^5^, ^6^, ^7^, ^8^, ^9^, ^10^, ^11^, ^12^, ^13^, ^14^, ^15^, ^16^, ^17^, ^18^, ^19^, ^20^, ^21^, ^22^, ^23^, ^24^, ^25^, ^26^, ^27^.

Some studies justify that the sensorineural hearing loss that happens with aging is related to a microcirculatory insufficiency that occurs due to vascular occlusion caused by emboli, hemorrhage or vasospasm, and these happen because of a syndrome of hyperviscosity or microangiopathy caused by diabetes or hypertension, and the latter could, through histopathological mechanisms cause the sensorineural hearing loss^28^, ^29^.

In an experimental study using rats with arterial hypertension, it was noted that hypertension is an important risk factor for age-related hearing loss. Action potentials, electrochemistry and potassium concentration in the cochlea of these genetically predisposed to hypertension animals were measured together with their normotension counterparts. With aging, the hypertensive animals had a higher action potential threshold, a higher electrochemical potential happened only in the extremely aged animals, while potassium concentration increased not only in the endolymphatic cells, but also in the perilymphatic ones. These data suggest that ionic modifications to the cell action potential are involved in the hearing reduction that happens to hypertensive animals. These data help us understand hearing loss in hypertensive individuals^7^.

It is known, however, that environmental factors, to which human beings may be subject, such as noise, inhalation of toxic substances, certain metabolic and circulatory alterations; infections, injuries of many natures and genetic inheritance, may also influence the individual's hearing, often times accelerating the process of cochlear degeneration^1^, ^2^, ^3^, ^4^, ^5^, ^6^, ^7^, ^8^, ^9^, ^10^, ^11^, ^12^, ^13^, ^14^, ^15^, ^16^, ^17^, ^18^, ^19^, ^20^, ^21^, ^22^, ^23^, ^24^, ^25^, ^26^, ^27^, ^28^, ^29^, ^30^, ^31^. In the present study, aiming at reducing selection bias problems, we excluded those individuals who worked in noisy environments, had diabetes, had previous history of hospital stay and ingestion of ototoxic drugs; notwithstanding, we know that in large urban centers there is intense exposure to environmental noise – depending on where the person lives, works, spends his leisure time, which may all provide excessive and long standing noise to the individual, thus accelerating hearing aging.

As to gender, there was a difference in the men to women ratio in both groups, due to the fact that we did not pair the sample – cases were taken at random, during regular medical visits, while the controls were brought by the hearing impaired patients themselves, under the instruction that they could not be siblings or parents of these patients, and we had to take off the study a greater number of men because of a hearing impairment suspicion – those who worked or had worked in noisy environments.

Many papers have approached the theme gender and hearing loss in relation to age^32^, ^33^. Dubno et al.^34^, after a study that correlated age, gender and hearing acuity for the spoken Word, reported that males had a significant age-related drop in their hearing acuity and speech recognition, while women did not show such pattern. Pearson et al.^35^, after a longitudinal study involving 681 men and 416 women, without signs of specific hearing disorders, unilateral or noise-induced hearing loss, reported that there is a two-fold increase in the speed at which men lose their hearing, when compared to women, showing that age and gender are indeed related to hearing loss even in groups without signs of noise-induced hearing loss.

Collet et al.^36^, after studying the influence of age and gender in ABR, reported that, using logistics regression it was possible to determine a correlation with hearing loss. In the present study, carried out with male individuals with ages varying between 45 and 64 years, the male gender (p=0.0004) proved to be an independent risk factor for hearing loss through the model of logistics regression we used. This corroborates the studies that have reported a significant drop in hearing acuity for male individuals as they age^20^, ^34^, ^35^, ^36^.

Due to a reduction in mortality rates, there is an increase in life expectancy all over the world. In Brazil, according to the recent census (2000), the elderly population corresponded to 5.85% of the population, and its growth was of 1.02% in relation to the previousscensus from the 90's. The aging index also increased from 13.90% in 1991 to 19.77% in 2000^20^.

Since medical development together with socioeconomical factors have drastically reduced the number of premature deaths, cardiovascular diseases and cancer are actually the main causes of death; and this is due not only to an increase in the incidence of these pathologies, but also to a greater longevity and mainly to medication control and efficacy in eliminating infectious diseases^37^, ^38^. The major factors that affect health today are the chronic diseases caused by genetics, life style, the environment and aging itself; thus, we should focus our attention on the last years of life of our patients aiming at increasing the number of health elderly, capable of maintaining their physical and mental functions until close to death^16^, ^37^, ^38^, ^39^, ^40^.

The new challenge are the chronic conditions related to aging, many detectable and preventable already in middle age, and such conditions are currently seen in the many health care specialties in delaying those. Fries sees this as “morbidity compression”, in other words, live a life with relative health and compress diseases to a short period of time right before death^41^. In order to achieve it, it is necessary to adopt principles and strategies for preventive care and health maintenance that should be particularly geared to each patient individually, aiming at improving their life quality. Among such preventive care, we should take care of the onset, as the many problems accruing from arterial hypertension, among which we mention hearing loss.

According to the Brazilian Council on Arterial Hypertension, high blood pressure is multicausal and multifactorial, because most of the time it does not cause any symptoms to the patients and it involves education aiming at achieving a number of goals, and for that there is the need to form a multidisciplinary care team^18^. The consensus also states that the multidisciplinary team can and should have processionals that are, in one way or the other, dealing with hypertensive patients. Physicians, nurses, nurse technicians, nutritionists, psychologists, social workers, community agents, physical education teachers, pharmaceutics and, administrative personnel should all be part of the team; and at no time they mention the speech and hearing therapist, who, according to article 442 have the competence to participate in diagnostic teams, carrying out the oral, voice, written and hearing assessment and collaborate in speech and hearing topics related to other sciences. Since arterial hypertension has proven to be a risk factor for hearing loss, and such onset should be checked in this population, the speech and hearing therapist, as the one responsible for hearing assessment, should be included as an effective member of such multiprofessional team involved with arterial hypertension.

The care related to arterial hypertension and hearing will certainly serve to avoid the frustrations caused by the reduction in one's capacity to understand oral language caused by a reduction in hearing accuity^39^, ^43^, ^44^, ^46^, which may happen to arterial hypertensive individuals.

## CONCLUSION

Since the study has shown that arterial hypertension is an independent risk factor for hearing loss, besides the male gender and advanced age, we highlight the importance of preventive processes that may mitigate the mechanisms that cause degeneration of the hearing apparatus caused by circulatory problems, most specifically high blood pressure.
